# Operative Standards for Cancer Surgery: Will Synoptic Reporting Really Improve Cancer Outcomes?

**DOI:** 10.1097/AS9.0000000000000440

**Published:** 2024-05-14

**Authors:** Ton Wang, H. Gilbert Welch, Lesly A. Dossett

**Affiliations:** From the * Department of Surgery, Duke University, Durham, NC; † Department of Surgery, Center for Surgery and Public Health, Brigham and Women’s Hospital, Boston, MA; ‡ Department of Surgery, University of Michigan, Ann Arbor, MI.

## INTRODUCTION

For most early-stage solid tumors, high-quality surgery represents the greatest potential for cure. While considerable attention has been given to the quality of diagnostic staging and adjuvant therapy, relatively little attention has been paid to the technical quality of cancer surgery. To address this gap, the American College of Surgeons (ACS) published 134 operative standards across 15 cancer types to improve surgical quality and outcomes.^1–3^ Based on data and expert opinion, these standards attempt to define critical elements of optimal cancer surgery.

Hoping to leverage these standards as a quality assurance tool for surgeons, the Commission on Cancer (CoC) implemented 6 standards in 2020. The CoC accredits over 1500 hospitals serving more than 70% of cancer patients in the United States. Given that the 6 standards target common cancers—breast, colorectal, lung, and melanoma—they potentially impact half a million operations annually. To maintain accreditation, CoC hospitals are expected to reach 80% compliance with operative standards by 2024.

## MECHANISM TO ASSESS COMPLIANCE: SYNOPTIC REPORTING

A crucial component of operative standards implementation is the mandatory use of synoptic reporting, in which key elements of the standard are self-assessed by the clinician and recorded in a checklist with prespecified data elements.^4^ Of the 6 standards that have been implemented to date, 4 require synoptic documentation from surgeons, and 2 require synoptic reporting from the pathology team. This standardization of operative and pathology reports is intended to provide a template for reporting the technical components of surgery and facilitate data collection, allowing the assessment of compliance, benchmarking, audits, and feedback.

How synoptic reporting is implemented at the hospital level, however, is far from standardized. The ACS allows hospitals to use 1 of several methods. Well-resourced hospitals may choose to develop homegrown systems integrated into their electronic medical records (EMRs); others may purchase off-the-shelf software packages and attempt to incorporate them into their EMRs. Those with fewer resources may revert to paper—using the PDFs available at the CoC website—and abstract the data manually. These processes must be considered in the context that quality metric reporting can be expensive and time-consuming.^5^ Thus, given the resources required to implement synoptic reporting, it is crucial to determine the relationship between operative standards and cancer outcomes before the standards are more broadly applied.

## POSSIBLE RELATIONSHIPS BETWEEN OPERATIVE STANDARDS AND CANCER OUTCOMES

Multiple scenarios are possible (**Table 1**):

**TABLE 1.** Possible Scenarios Following the Introduction of Synoptic Reporting

Compliance With Operative Standards	Cancer Outcomes	Scenario	Assessment of CoC Operative Standards
Improved	Improved	Compliance with operative standards results in improved cancer outcomes	Effective
Improved	Improved	Cancer outcomes are improving over time independently of operative standards	Ineffective
Improved	Spuriously improved	Stage-specific cancer outcomes improve as a result of upstaging secondary to more extensive lymph node sampling with no change in overall cancer outcomes	Ineffective
Improved	No change	Operative standards are unrelated to cancer outcomes	Ineffective
No change	Improved or no change	Operative standards are either already being met or are being ignored	Ineffective

### Compliance with operative standards improves, which results in improved cancer outcomes

Synoptic reporting may cue surgeons to the standards of high-quality cancer surgery, and meeting those standards may genuinely improve oncologic outcomes (eg, lead to more appropriate selection of surgical treatment, improve completeness of disease resection, or improve accuracy of nodal staging, which in turn could result in more appropriate multidisciplinary delivery of adjuvant therapies that improve cancer outcomes.)

### Compliance with operative standards improves, as do cancer outcomes, but the two are independent

In general, cancer outcomes improve over time. Failure to account for temporal trends in treatment and diagnostic practice when assessing the utility of operative standards could lead to an erroneous conclusion that operative standards were effective.

### Compliance with operative standards improves, which results in spuriously improved cancer outcomes

Standards targeting the adequacy of lymph node sampling may result in more extensive sampling and stage migration: patients who previously would have been classified in a lower stage are now being assigned to a higher stage. This upstaging leads to improved stage-specific survival at each stage, even if overall survival is unchanged (ie, Will Roger’s effect).^6^

### Compliance with operative standards improves, but there is no change in cancer outcomes

Some operative standards may target outcomes that cannot be appreciably improved (ie, a floor effect: outcomes with extraordinarily low event rates, such as local recurrence after nonhead and neck melanoma resection with guideline-concordant margins).^7^ Others may be spuriously improved (a surgeon attests to removing all palpable nodes, but palpable nodes remain). Finally, some standards may improve but be unrelated to cancer outcomes (eg, reporting the preservation of sensory and motor nerves during axillary lymph node dissection).

### Compliance with operative standards is unchanged

Some standards may be already performed at high rates (ie, ceiling effect).^7^ Alternatively, surgeons may choose not to incorporate the standards into their practice. In this scenario, any change in cancer outcomes would be independent of the operative standards.

## A CALL FOR RIGOROUS EVALUATION

The operative standards represent a major and commendable investment by the ACS and CoC to improve surgical quality and evidence-based cancer care. These standards were designed with input from key stakeholders and the public, with iterative revisions before implementation, with the expectation of improving cancer outcomes in the United States. Implementation of the operative standards will likely result in intangible benefits through synoptic reporting and improved documentation, better coordination of multidisciplinary care, and broad attention to improving surgical quality. However, the impact of the operative standards on measurable oncologic outcomes, including overall survival, disease-specific survival, and cancer recurrence, should be rigorously evaluated. The ongoing implementation of operative standards should be leveraged to understand not only their impact on cancer outcomes but also their impact on surgeons and hospitals. We suggest the following:

For the 6 recently implemented standards, robust inference on their effect will be challenging as the rollout involves all CoC hospitals with no patients or hospitals serving as a control group. Nevertheless, interrupted time series analyses can be used to measure cancer outcomes before and after operative standards were implemented, while accounting for underlying temporal trends (which typically move in favor of improved outcomes). What can be robustly determined, however, is how diverse hospitals ultimately implement synoptic reporting across multiple operations and the costs involved. This should provide useful information to guide the rollout of future standards and their corresponding synoptic reporting requirements.

For subsequent standards, more robust inferences of the effect of operative standards are possible, and the implementation of standards should be guided by the need for their rigorous evaluation. Specifically, the implementation of subsequent standards should be randomized. For example, CoC hospitals could be randomized to be early or late adopters of all new standards, in which the late adopters serve as the control for the early adopters. Alternatively, hospitals could be randomized to implement some standards but not others (eg, 3 new standards are implemented early in 1 group of hospitals and 3 in the other). Given that all CoC hospitals submit data to the National Cancer Database (NCDB), these implementation designs offer a pragmatic opportunity to evaluate the effect of operative standards on many cancer outcomes. For outcomes that are not currently collected as a part of National Cancer Database, the CoC special study mechanism can be leveraged for rapid feedback on specific cancer outcomes.

If operative standards improve outcomes following cancer surgery, they should be broadly implemented. If, on the other hand, they are found to be ineffective, they should be revised. Any revisions should reengage key stakeholders in discussions both about the utility of the proposed standard and the barriers to the implementation of synoptic reporting.
